# Harlequin color change in a premature infant: A case report

**DOI:** 10.1002/ccr3.7840

**Published:** 2023-08-24

**Authors:** Linzhi Yang, Guiying Zhuang, Dahu Zhang, Weiqi Liu

**Affiliations:** ^1^ Department of Neonatology The Maternal and Children Health Care Hospital (Huzhong Hospital) of Huadu Guangzhou China; ^2^ Department of Clinical Laboratory The Maternal and Children Health Care Hospital (Huzhong Hospital) of Huadu Guangzhou China

**Keywords:** blood extraction, compensatory acidosis, harlequin color change, midazolam, neonate

## Abstract

**Key Clinical Message:**

This case of HCC report contributes to the knowledge of HCC in China. In this case, the longer duration of the color change observed in this case compared to previous reports, which will be useful for all medical practitioners.

**Abstract:**

Harlequin color change (HCC) is a benign skin color change that lasts for a short time with no obvious physical abnormalities. Its pathogenesis is still unclear. It occurs in newborns, especially premature infants. However, few cases of HCC have been reported in China. Herein, we report a case of HCC. The infant was born at 34 + 4 weeks of gestation and was admitted to the hospital due to metabolic acidosis and neonatal pneumonia after birth. On the third day after birth, there were two red bands with obvious edges along the body centreline, and the erythema characteristics were consistent with those of HCC. The immature hypothalamus of newborns may cause the occurrence of HCC. At the same time, some drugs (midazolam), hypoxemia, and blood sampling may also be associated with HCC during neonatal hospitalization. All doctors should be thoroughly knowledgeable about the clinical characteristics of HCC and avoid using unnecessary drugs during treatment.

## Graphical abstract

Harlequin color change (HCC) Changes along Midline.

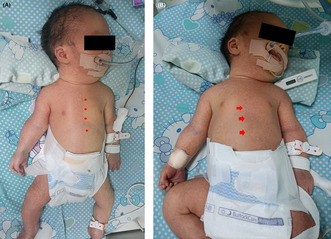

## 1 INTRODUCTION

Harlequin color change (HCC) is a rare, benign, and transient color change that often occurs in newborns. When the disease occurs, the body often appears half red and half pale.^1^ Since the first case was reported in 1952,^2^ reports of HCC have also increased gradually. It has been reported that the incidence of HCC in newborns is as high as 10%,^3^ and premature infants are more prone to HCC than full‐term infants.^4^ The results showed that the occurrence of HCC is closely related to the immature hypothalamus.^2^, ^5^ In a subsequent study, it was shown that prostaglandin E1,^6^ hypoxemia,^7^ and invasive manipulation^8^ may also be important reasons for the occurrence of HCC. Reports on HCC mainly come from developed countries, but there are relatively few reports in China. In this report, we described a case of HCC in a premature child with neonatal pneumonia in Guangzhou, China.

## 2 CASE REPORT

### 2.1 The neonate

A male newborn was born at 34 + 4 weeks after cesarean section due to malposition, with a birth weight of 2700 g and Apgar scores of 8, 9, and 10. On admission, the infant presented with dyspnea, and digital radiography of the chest revealed neonatal pneumonia. After admission, the infant showed decreased blood pressure, marbling throughout the body, a capillary refill time of 3 s, pale skin, cold limbs, low pulse pressure, and physical manifestations consistent with neonatal shock signs. Therefore, the infant was given mechanical respiratory ventilation, anti‐infective drugs, low‐dose dopamine to improve circulation, and midazolam sedation. Echocardiography revealed patent ductus arteriosus (2.1 mm), patent foramen ovale (2.7 mm), tricuspid regurgitation (mild), and normal cardiac function. The results of ambulatory electroencephalography showed that the background activity was continuous, sleep–wake cycles were observed, and no abnormal spikes or convulsions were found. The results of head magnetic resonance imaging showed no abnormalities. The results of blood, cerebrospinal fluid, and gastric fluid were normal.

**FIGURE 1:**
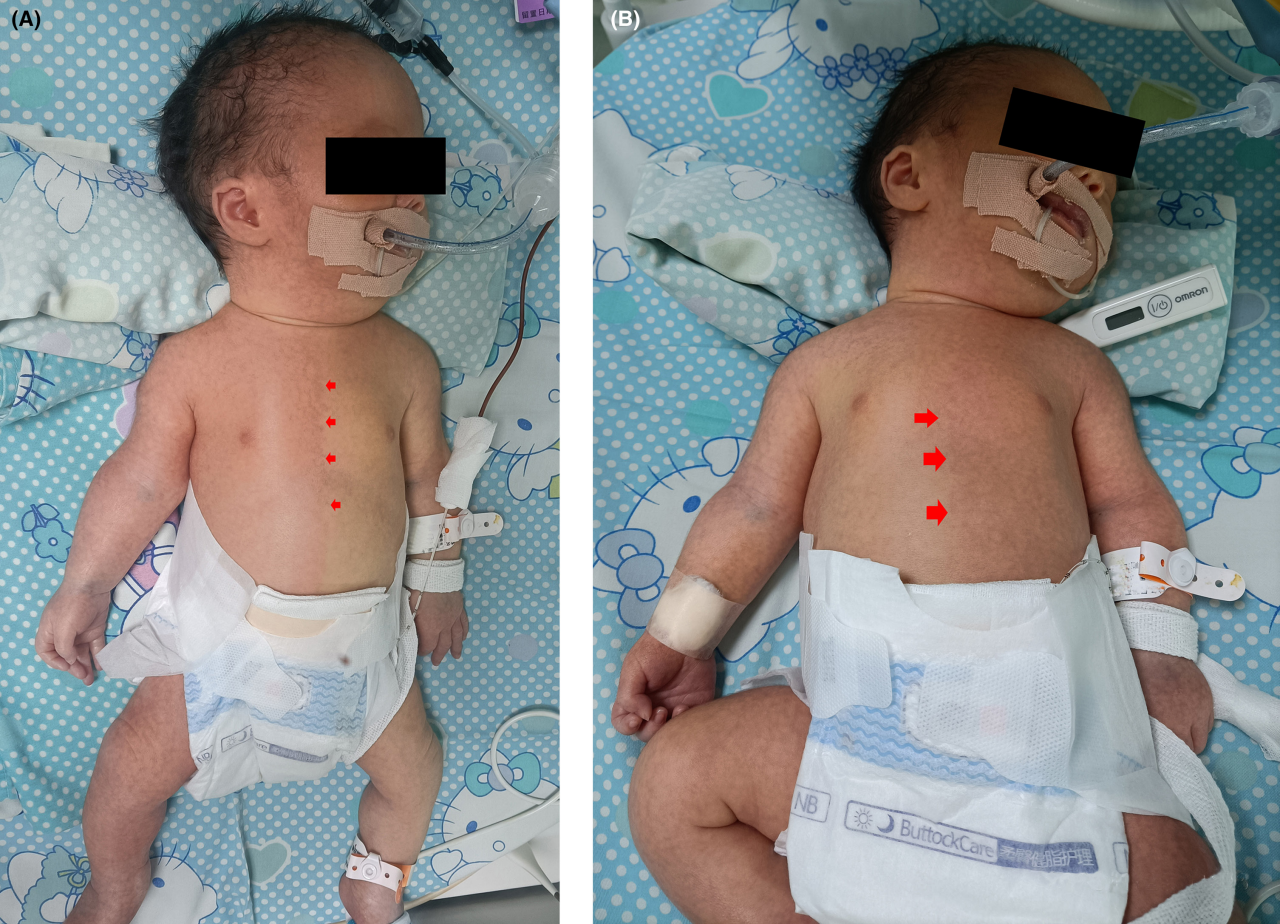
(A) Body color changes included diffuse redness on the right side and paleness on the left side of the body, with a clear midline from the face to the lower limbs. (B) Body color changes included diffuse redness on the left side and paleness on the right side of the body, with a clear midline from the face to the lower limbs.

On the third day, the b*lood gas analysis* results showed mild compensatory acidosis, and well‐defined erythema appeared on the right side of the body after midazolam and dopamine were stopped for 3 h and 2 h, respectively (Figure 1A), lasting approximately 60 min. When the erythema on the right side of the body disappeared, venous blood was collected for physical health monitoring. Approximately 30 min after resolution of the first case of erythema, an erythematous area with the same clear boundary appeared on the left side of the body (Figure 1B), lasting for approximately 30 min. During the period of erythema, the vital signs were stable, and the characteristics of this redshift were consistent with those of HCC. No similar phenomenon occurred after a follow‐up of 1 month.

### 2.2 The neonate's mother

The boy's mother was 35 years old, was not married to a close relative, had no history of infectious diseases, and had a history of pregnancy. This case occurred in her second infant. No dexamethasone was used to promote lung maturation before delivery, and no other drugs were used. The first child was healthy and never experienced HCC.

## 3 DISCUSSION

The first HCC report showed that the younger the gestational age, the more likely HCC is to occur, especially in premature infants,^2^ as verified in subsequent reports.^9^, ^10^, ^11^ The etiology and pathogenesis of HCC are still unclear. Most scholars believe that HCC occurs due to sympathetic dysfunction in tension control of the peripheral capillary bed caused by the immature hypothalamus.^6^, ^12^, ^13^ This patient was a premature infant, which may be one of the reasons for the occurrence of HCC due to the dysfunction of peripheral vascular control caused by the immature hypothalamus.

The diagnosis of HCC is mainly based on the change in the skin color of specific parts of the body, with clear boundaries. The duration of onset varies from 30 s to 20 min, and the color can automatically return to normal, while the vital signs remain stable.^1^, ^14^ HCC usually occurs from the third to fifth day after birth.^3^, ^8^, ^15^ This case occurred on the third day after birth, and compared to previous reports of HCC duration lasting seconds to 15 min,^8^, ^11^, ^16^ the current episode shows a significantly longer duration, with a maximum duration of 60 min. This finding will be beneficial for all medical practitioners, especially those involved in neonatal care, should be familiar with this phenomenon, and avoid using unnecessary drugs during treatment.

It has been reported that infant position changes are an important factor causing HCC, and position changes can cause HCC,^1^, ^17^, ^18^ which is not consistent with this case. There was no change in the body position of the child before, during, or after the occurrence of HCC. However, hypoxia may trigger the pathogenesis of HCC^15^; the infant had respiratory disorders in the early stage, which may be related to the occurrence of HCC.

In most cases, newborns with HCC show a sudden change in skin color, that is, there is a red band with obvious edges along the body centreline,^10^ which is consistent with this study. In this case, there were two red bands with obvious edges along the midline of the body that developed in a short time. Some studies have shown that hypoxemia and midazolam can enhance the expression of HCC,^18^ which is consistent with the first occurrence of HCC in this infant. The child was in compensatory acidosis before the onset of the first occurrence of HCC, and the use of midazolam might have affected the tension of the peripheral capillaries, leading to the occurrence of the first HCC. Dand et al.^11^ showed that blood extraction is also one of the possible causes of HCC. A venous blood draw was performed before the onset of the second occurrence of HCC and may have been its main cause.

In summary, HCC is a benign skin color change with a short duration and no significant physical abnormalities. Since there have been few reported cases of HCC in China, this case report adds to the knowledge about HCC in the country. Additionally, the duration of color change observed in this case is longer than previously reported cases, which highlights the importance of re‐evaluating the duration of HCC. Furthermore, this self‐limiting phenomenon helps in the differential diagnosis of other diseases, such as papular rash and urticaria. After the occurrence of HCC, it may be of great help to explain the condition to the parents of the infant.

## AUTHOR CONTRIBUTIONS

**Linzhi Yang:** Validation; writing – original draft. **Guiying Zhuang:** Investigation; validation; writing – original draft. **Dahu Zhang:** Investigation; validation. **weiqi Liu:** Conceptualization; investigation; project administration; supervision; writing – original draft; writing – review and editing.

## CONFLICT OF INTEREST STATEMENT

The authors declare that the research was conducted in the absence of any commercial or financial relationships that could be construed as a potential conflict of interest.

## ETHICS STATEMENT

This study was approved by the Ethics Committee of the Maternal and Child Health Hospital of Huadu District (No. 2023–005).

## CONSENT STATEMENT

Written informed consent was obtained from the patient's parents to publish this report in accordance with the journal's patient consent policy.

## Data Availability

None.
